# Sensitivity of the human auditory cortex to acoustic degradation of speech and non-speech sounds

**DOI:** 10.1186/1471-2202-11-24

**Published:** 2010-02-22

**Authors:** Ismo Miettinen, Hannu Tiitinen, Paavo Alku, Patrick JC May

**Affiliations:** 1 Department of Biomedical Engineering and Computational Science, Aalto University School of Science and Technology, Espoo, Finland; 2 Department of Signal Processing and Acoustics, Aalto University School of Science and Technology, Espoo, Finland; 3 BioMag Laboratory, Hospital District of Helsinki and Uusimaa HUSLAB, Helsinki University Central Hospital, Helsinki, Finland

## Abstract

**Background:**

Recent studies have shown that the human right-hemispheric auditory cortex is particularly sensitive to reduction in sound quality, with an increase in distortion resulting in an amplification of the auditory N1m response measured in the magnetoencephalography (MEG). Here, we examined whether this sensitivity is specific to the processing of acoustic properties of speech or whether it can be observed also in the processing of sounds with a simple spectral structure. We degraded speech stimuli (vowel /a/), complex non-speech stimuli (a composite of five sinusoidals), and sinusoidal tones by decreasing the amplitude resolution of the signal waveform. The amplitude resolution was impoverished by reducing the number of bits to represent the signal samples. Auditory evoked magnetic fields (AEFs) were measured in the left and right hemisphere of sixteen healthy subjects.

**Results:**

We found that the AEF amplitudes increased significantly with stimulus distortion for all stimulus types, which indicates that the right-hemispheric N1m sensitivity is not related exclusively to degradation of acoustic properties of speech. In addition, the P1m and P2m responses were amplified with increasing distortion similarly in both hemispheres. The AEF latencies were not systematically affected by the distortion.

**Conclusions:**

We propose that the increased activity of AEFs reflects cortical processing of acoustic properties common to both speech and non-speech stimuli. More specifically, the enhancement is most likely caused by spectral changes brought about by the decrease of amplitude resolution, in particular the introduction of periodic, signal-dependent distortion to the original sound. Converging evidence suggests that the observed AEF amplification could reflect cortical sensitivity to periodic sounds.

## Background

Speech perception is an intricate process exceptionally resilient to distortions of almost any kind, whether occurring in natural environments or caused by manipulation of particular stimulus features in laboratory conditions. This extraordinary robustness enables successful communication under acoustically adverse conditions. Despite decades of research and development, attempts to create artificial speech recognition systems have demonstrated that the human brain is far superior to machines in interpreting speech signals in noisy or otherwise degraded conditions [1]. How the brain achieves this computational feat, however, has remained unsolved.

The basis of the robustness of human speech perception lies in the fact that there does not seem to be a single indispensable property within the acoustic signal upon which the entire recognition process relies. This view is supported by a wealth of behavioral research aimed at delineating the boundary conditions of speech intelligibility. Drullman [2], for example, demonstrated that speech perception does not depend on the extraction of fine structure cues present in the temporal envelope by showing that an intact temporal speech envelope with random fine structure retains perfect intelligibility. Further, Loizou et al. [3,4] established that when there are enough spectral channels available, a relatively small spectral or temporal amplitude resolution is sufficient for successful recognition of speech. Then again, also the spectral information in speech contains significant redundancies, as documented by Shannon et al. [5], who showed that speech recognition is possible even with very few spectral cues as long as temporal envelope cues are present in a few contiguous spectral regions. Moreover, Saberi and Perrott [6] demonstrated that continuous speech is highly resistant to time reversal of local segments up to a duration of 50 ms, which indicates that a detailed analysis of the short-term acoustic spectrum is not essential for speech comprehensibility.

In natural auditory environments, speech signals can be subjected to various kinds of external, "additive" distortions. These distortions can be caused, for example, by different types of environmental noise or informational masking by several concurrent speakers. Although the amount and quality of temporal and spectral cues of speech can be reduced considerably without a significant decrease in recognition performance, the inclusion of external noise poses an additional challenge to the perceptual mechanisms [7]. Based on behavioral results, it appears that the spectral structure of external masking noise has a profound effect on the efficiency of the noise as a speech masker. For example, Studebaker et al. [8] found that noise which was spectrally matched to the speaker's voice spectrum was a significantly more effective masker than uncorrelated white or high-pass noise.

The neural processing of intact as well as distorted speech has recently aroused interest within the cognitive neurosciences. Given that both the perceptually significant alterations in acoustic speech signals and the ensuing cognitive processes in the brain take place on a timescale of milliseconds, the high temporal resolution of electro- (EEG) and magnetoencephalography (MEG) is ideal for measuring the rapid changes in brain activation during speech perception. The prominent N1m wave of the auditory evoked field (AEF), in particular, has been shown to exhibit sensitivity to a variety of acoustic attributes of the speech signal [9-15].

A majority of earlier studies aimed at investigating cortical responses to degraded speech have used continuous stochastic noise to distort the speech stimuli. Stochastic noise has a random frequency spectrum which does not correlate with the spectral structure of the speech signal. In these studies, the noise was presented continuously in the background, resulting in an elevated recognition threshold for the speech stimuli as the signal-to-noise ratio was decreased. Using continuous low- and high-pass stochastic noise, Martin et al. [16,17] demonstrated that the N1, N2, and P3 responses elicited by speech sounds were delayed and reduced in amplitude as the bandwidth of the masking noise was increased. In a related study, Whiting et al. [18] showed that when speech sounds were distorted with broadband stochastic noise, the auditory evoked potential (AEP) amplitudes were attenuated and latencies increased as the signal-to-noise ratio was reduced. Corroborating results were obtained by Muller-Gass et al. [19], who showed that the AEPs were decreased in amplitude and increased in latency as the intensity of the speech-masking noise was raised. Furthermore, Kozou et al. [20] studied the effects of different background noise types on brain responses to speech and non-speech stimuli and discovered similar decrements in AEP amplitudes with decreasing signal-to-noise ratios. While the various noise types yielded somewhat different results, their effect was qualitatively the same, i.e., a decrease in the amplitude of the brain response. Further evidence that stochastic noise results in delayed and attenuated evoked brain responses to speech sounds was demonstrated also with noise that initiated one second prior to the speech stimulus instead of being continuous throughout the measurement [21]. Similar increases in AEP latencies with decreasing signal-to-noise ratio were found. The effect of noise on the AEP amplitudes was less systematic, although some of the stimuli elicited a decreasing trend of the N1 amplitude with the reduction of the signal-to-noise ratio [21]. A number of MEG studies have also revealed hemispheric asymmetries using white-noise maskers. For example, Shtyrov et al. [22,23] found that white noise depresses left-hemispheric AEFs to speech sounds while right-hemispheric AEFs remain constant or even increase in amplitude. Taken together, most of the neurocognitive studies investigating the effects of external distortions of speech have employed only maskers whose spectra do not correlate with the intact speech spectrum. However, given that the strength of spectral correlation between the original signal and the masker considerably affects speech intelligibility on the perceptual level (see, e.g., [8]), it seems possible that this effect could be observed in brain dynamics as well.

In addition to external noise, speech intelligibility can be compromised by directly manipulating the acoustic structure of the speech signal. A recent study of ours [24] demonstrated that degrading speech by using a method that results in spectrally correlated, signal-dependent distortion yields different effects on brain activation than those reported in previous studies using additive, uncorrelated stochastic masking. The signal-dependent distortion was generated with uniform scalar quantization (USQ), a method for degrading auditory signals based on time-domain sampling and representing the amplitude values of the temporal waveform on a finite scale (see, e.g., [25]). Essentially, the quantization decreases the amplitude resolution of the signal waveform, yielding a significant reduction in speech intelligibility. The results showed that activation in the right-hemispheric auditory cortex as measured through the N1m amplitude increased when the amount of signal-dependent distortion was raised. Furthermore, the depression of left-hemispheric AEFs with increasing noise level reported by Shtyrov et al. [23] was not observed. These results, which are at odds with the findings discussed above [16-23], are interesting considering that the distortion we used reduces the comprehensibility of speech very effectively as well. The diverging results are further accentuated by the fact that both quantization and uncorrelated white noise flatten the spectrum of speech by adding high frequencies to the original signal. Moreover, both distortions increase the spectral bandwidth of spectrally simple, narrowband sounds. Consequently, the most conspicuous spectral difference between quantized and white noise -masked sounds is that in the former the degradation process generates new, noisy spectral components that are located mainly at the harmonics of the original, intact sound spectrum. In other words, the spectral structure of the quantized sound, in comparison to its white-noise corrupted counterpart, correlates more closely with the spectrum of the original signal. As the differential results are presumably due to these distinctions between signal-dependent distortion and uncorrelated noise utilized in the studies, it seems that the effects of stimulus degradation on brain responses are highly dependent on the characteristics of the distortion.

One might speculate that the previously demonstrated increase in the N1m amplitude is related to processing of acoustic properties of speech in particular [24]. Alternatively, because speech processing is classically thought of as being lateralized to the left hemisphere, the observed right-hemispheric sensitivity could be related to processing of basic acoustic features of all sounds instead of speech-specific processes. Given that the quantization procedure substantially modifies the spectral structure of speech, the latter interpretation gains support from studies showing that the right-hemispheric auditory cortex is more sensitive to spectral variation of sounds than the left [26-28]. In the current study, our aim was to determine whether the increase of activation in the right-hemispheric auditory cortex caused by the signal-dependent distortion can be observed also with spectrally simpler non-speech sounds. In order to link brain measures with behavioral data, an identification experiment was conducted to find out whether the non-speech sounds are perceptually separable from the speech sounds when the amount of degradation is increased.

## Methods

### Participants

Sixteen right-handed volunteers (average age 26.6 years; SD = 5.27; 9 male) took part in the study with informed consent. All were native Finnish speakers with normal hearing. The experiment was approved by the Ethical Committee of the Helsinki University Central Hospital.

### Stimuli

The stimuli comprised the Finnish vowel /a/, a complex non-speech sound, and a sine-wave tone. The vowel was created by using the Semi-synthetic Speech Generation method, which enables the production of fully controlled natural-sounding speech stimuli [29]. Firstly, a waveform of the vowel /a/ was created from a natural utterance of a Finnish male speaker (F0 = 113 Hz) by estimating the glottal excitation pulseform produced by the vibrating vocal folds. Secondly, this waveform was used as input to an artificial vocal tract modeled by a digital all-pole filter. The complex non-speech sound was a composite of sinusoids created by combining five sine-wave tones whose frequencies were matched to the major spectral harmonics of the vowel /a/: the first frequency (113 Hz) was adjusted to be equal to the fundamental of the speech spectrum, the other four tone frequencies (565, 904, 1933, & 3435 Hz) were selected to coincide with the strongest harmonics in the vicinity of the lowest four formants of the vowel spectrum. The frequency of the simple sine-wave tone (565 Hz) coincided with the strongest individual harmonic in the entire vowel spectrum, that is, the fifth harmonic in the vicinity of the first formant.

**Figure 1:**
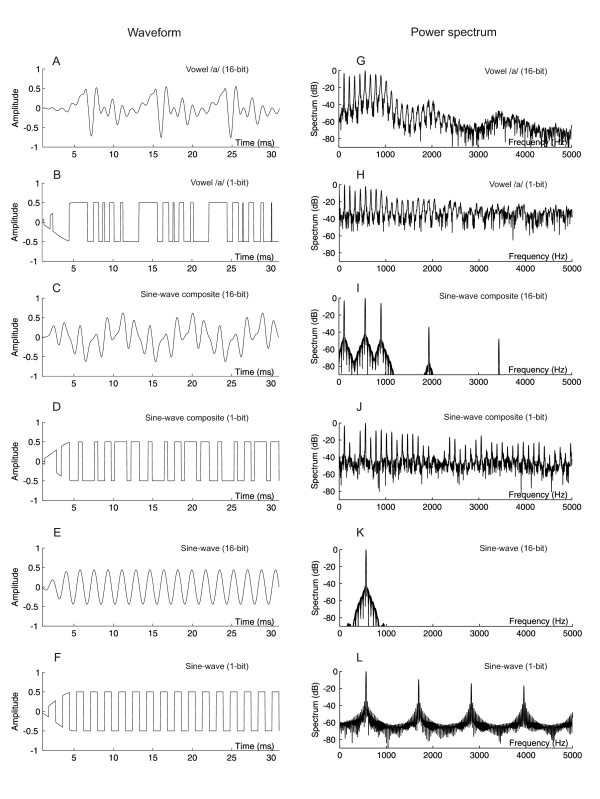
**The effect of stimulus manipulation using uniform scalar quantization**. Left column (A-F): the 16-bit and 1-bit waveform of each stimulus type. Right column (G-L): the power spectrum of 16-bit and 1-bit mode of each stimulus type.

In order to degrade the synthesized sounds in a controlled manner, each signal was modified with uniform scalar quantization. Degradation was implemented stepwise by reducing the number of bits in the sample quantization from 16 to 4 to 1. The differences between the 16-bit and 1-bit temporal waveforms and power spectra of the stimuli are shown in Fig. 1. A 16-bit signal, having 65536 levels to quantify sound amplitude, is perceptually equivalent to an analog signal of the same bandwidth. Decreasing the bit mode reduces the amplitude resolution of the temporal waveform, and this is manifested in the frequency domain as an increase in quantization noise (cf [4,24]). The generation of distortion by the USQ method is, importantly, very different from that based on merely adding (white) noise to an undistorted speech signal. Namely, in quantizing the original sound sample, the USQ method acts as a non-linear process. Hence, as described by the theory of signals and systems [30], it creates *new *frequency components into the input signal, a feature which is not possible with linear filtering. In addition, since the USQ method reduces the amplitude resolution of the input signal, the flatness of the output spectrum will be increased. Taken together, these two factors imply that if the input signal comprises only a single frequency component, the output spectrum will be flat but will consist of noisy harmonics of the input frequency (Fig. 1L). If the input has several harmonics, as in the case of a tone composite, each component will generate new harmonics in the USQ process, and the output spectrum will have a flat overall trend with a dense structure of noisy harmonics (Fig. 1J). In the case that the input signal is a vowel comprising a large number of harmonics over a wide frequency range, the output spectrum will again be flat comprising noisy harmonics that are located at the multiples of the F0 of the input (Fig. 1H). We note that the spectral structure of the degraded vowel (Fig. 1H) resembles that of the undistorted speech sound (Fig. 1G) whereas a much larger relative spectral change is caused by signal degradation in the case of, for example, sinusoidal composites (Figs. 1I and 1J). In summary, by utilizing three types of periodic input signals (sinusoidal, composite of sinusoidals, vowel), all of which are spectrally unique but share the feature of including spectral harmonics, the USQ process can be used to study processing of sound degradation which is *signal-dependent*.

All the stimuli were 200 ms in duration and matched in rise/fall times (5 ms). The stimuli were presented binaurally through a pair of plastic tubes and ear pieces. Sound intensity was measured at the ear pieces using a sound level meter (Velleman DVM 805) and this value was adjusted to 75 dB(A) for each stimulus. This intensity calibration was conducted using the standardized A-frequency weighting together with standardized F-time weighting in which the SPL is integrated as the root mean square (RMS) value within a 125 ms time window. The stimuli were arranged in separate sequences presented in a randomized order counterbalanced across the participants. The onset-to-onset inter-stimulus interval was 1000 ms.

### MEG registrations

Auditory evoked fields (AEFs) were recorded in a magnetically shielded room with a 306-channel whole-head MEG device (Vectorview 4D, Neuromag Oy, Finland). Cortical activation was sampled at 0.6 kHz and band-pass filtered at 0.01-200 Hz before online averaging in a 600-ms window including a 100-ms pre-stimulus baseline. Epochs in which the magnetic signal exceeded an absolute amplitude variation of 3000 fT/cm were discarded online. Eye-movement artefacts were monitored by two electrode pairs and automatically excluded during the registration (threshold = 150 μV). A minimum of 150 artefact-free epochs were sampled for each stimulus. Before the acquisition, the locations of the head-position indicator coils were determined using a three-dimensional digitizer. The head-based coordinate system was defined by the *x*-axis passing through the preauricular points (positive to the right), the *y*-axis passing through the nasion, and the *z*-axis as the vector cross product of the *x- *and *y- *unit vectors. The head-position indicator coil locations were determined before each stimulus block. During the acquisition, the participant was seated in a reclining chair and was under instruction not to pay attention to the auditory stimuli and to watch a self-selected silent movie.

### MEG data analysis

The auditory N1m was studied for effects in response amplitude, latency and source location. A 100-ms pre-stimulus baseline and a filter passband of 1-30 Hz were used in calculating the AEF gradients. To quantify the cortical activity in the left and right hemispheres, unrestricted equivalent current dipoles (ECDs, see [31]) were determined using a set of 44 planar gradiometers over each temporal region. The ECDs were fitted to a single time point defined as the maximum dipole moment in the interval 85-140 ms. The left-hemispheric data of five subjects were discarded from the ECD analysis due to poor signal-to-noise ratio, with goodness-of-fit values <60% or with anomalous generator locations. The average goodness-of-fit values of the ECDs accepted for further analyses was 90.3%.

In addition to ECD analyses, the peak amplitudes and latencies of the P1m, N1m and P2m waves of the AEF were quantified from the pair of gradiometer channels exhibiting maximal vector sum amplitudes. This analysis, carried out separately for each hemisphere, allowed us to investigate the effects of stimulus distortion specifically on the P1m and P2m responses, for which ECD source modeling could not be reliably performed.

### Behavioral experiment

We tested the subject's ability to identify the USQ-degraded stimuli as either a particular vowel or as a non-vowel. The stimuli consisted of five Finnish vowels  (/a/, /e/, /i/, /o/ and /u/), created with the technique described in Section 2.2, as well as sine-wave composites and sine-wave tones, each quantized to 16-bit, 4-bit and 1-bit modes. In a forced-choice task, the subject was instructed to respond to each stimulus with a keyboard stroke. The response alternatives comprised all eight of the Finnish vowels and a "not-a-vowel" category. Since the 16-bit mode is perceptually equivalent to an analog signal, it was expected that all of the 16-bit stimuli would be easily identifiable. The stimuli were presented through headphones in a randomized order, with 10 repeats per stimulus. Identification accuracy and reaction times for each stimulus were determined by calculating the average accuracy and reaction time of the responses.

### Statistical analyses

In the MEG experiment, the amplitude, latency and source location of the N1m were analyzed with repeated-measures analyses of variance (ANOVA) for effects of hemisphere, stimulus type, and bit mode. In the behavioral experiment, the reaction times and identification accuracy were analyzed with ANOVA for effects of stimulus type and bit mode. The Newman-Keuls test was used in all post-hoc comparisons.

## Results

### N1m ECD modeling

**Figure 2:**
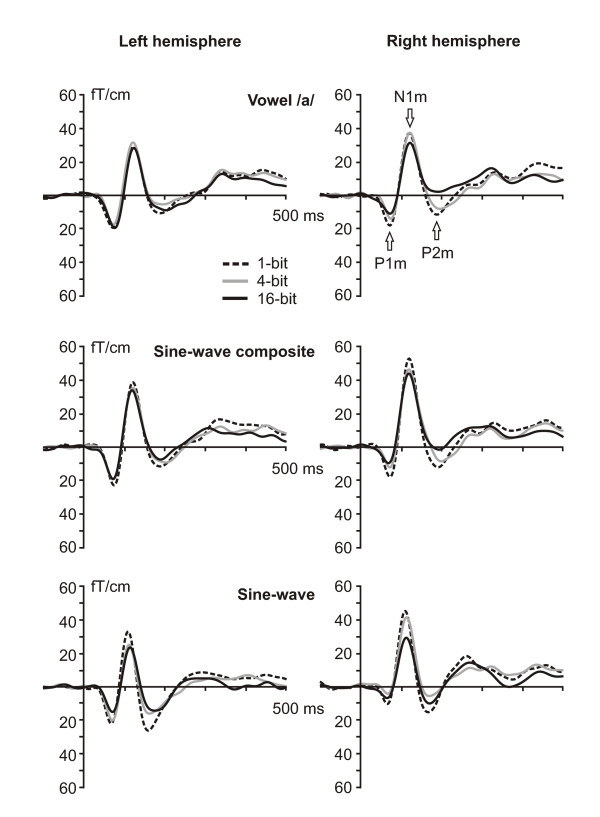
**The effect of stimulus degradation on the auditory evoked fields**. Grand-averaged waveforms for all stimuli from MEG gradiometer channels with maximum response amplitude in the left and right hemisphere.

**Figure 3:**
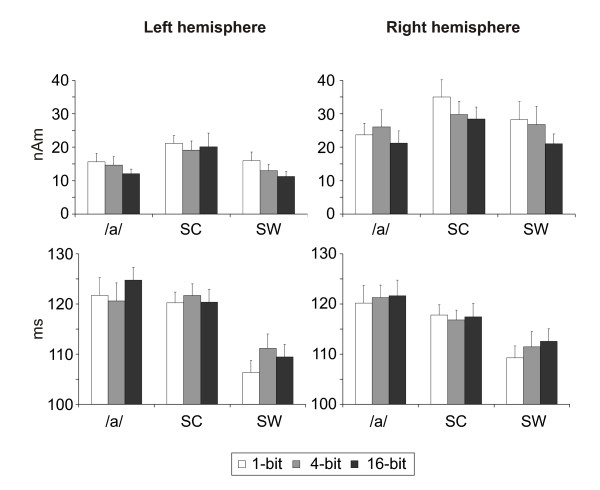
**The effect of stimulus degradation on the N1m ECD amplitude and latency**. The effects of bit mode on the average ECD amplitude and latency of N1m for each stimulus category (/a/:  vowel /a/; SC: sine-wave composite; **SW**: sine-wave). Error bars indicate SEM.

All stimulus types elicited prominent AEF responses in both hemispheres, as depicted in Fig. 2. The N1m amplitudes and latencies obtained through ECD modeling are shown in Fig. 3.

#### N1m amplitude

The grand-averaged N1m responses were larger in the right hemisphere (21.3 - 35.0 nAm) than in the left (11.2 - 21.1 nAm) [F(1,10) = 5.42, P < 0.05]. Importantly, the degradation of the stimuli (i.e., the decrease in bit level) resulted in a significant increase of N1m amplitude for all stimulus types, ranging from 19.0 nAm in the 16-bit mode to 23.3 nAm in the 1-bit mode [F(2,20) = 4.50, P < 0.05]. The overall amplitude of the N1m was dependent on stimulus type, with the sine-wave composite eliciting, on average, a stronger response (25.6 nAm) than the sine-wave tone (19.4 nAm) and the vowel stimuli (18.9 nAm) [F(2,20) = 11.65, P < 0.001]. No statistically significant interactions between any of the factors (hemisphere, bit mode and stimulus type) were observed.

When the ECD amplitudes of the N1m were analyzed separately for each hemisphere, an unexpected effect of bit mode was found in the left hemisphere, where a decrease in the bit level amplified the N1m response [F(2,20) = 4.29, P < 0.05]. The left-hemispheric N1m amplitude was also dependent on stimulus type [F(2,20) = 6.19, P < 0.01], the sine-wave composite eliciting a stronger response (20.1 nAm) than the other stimulus types (13.4-14.1 nAm for tone and speech stimuli, respectively). The stimulus type × bit mode interaction was not statistically significant for the left-hemispheric N1m amplitude. In the right hemisphere, similar results were obtained: the sine-wave composite elicited a stronger N1m (31.1 nAm) than the other stimuli (23.6-25.4 nAm for tone and speech stimuli, respectively) [F(2,30) = 8.88, P < 0.001] and the reduction in bit mode resulted in an amplification of the N1m response (23.6, 27.5 & 29.0 nAm for the 16-bit, 4-bit and 1-bit mode, respectively) [F(2,30) = 5.22, P < 0.05]. No significant stimulus type × bit mode interaction was observed.

#### N1m latency

No differences between the left and right hemisphere were observed in the N1m latency [F(1,10) = 0.13, P = n.s.]. The sine-wave tones elicited, on the average, N1m responses 10 ms earlier than spectrotemporally more complex stimuli (122, 119 & 110 ms for the speech sounds, sine-wave composites, and tones, respectively) [F(2,20) = 36.47, P < 0.001]. Furthermore, the reduction of bit level resulted in a monotonic decrease of N1m latency [F(2,20) = 4.11, P < 0.05]. Post-hoc tests revealed that this effect was caused by sine-wave tones eliciting the N1m response approximately 4 ms earlier for the 1-bit mode (107 ms) than the higher bit modes (111 ms). Separate analyses for each hemisphere yielded a stimulus type - bit mode interaction on N1m latency in the left hemisphere [F(4,40) = 3.95, P < 0.01]. Compared to the N1m elicited by the sine-wave composite and speech stimuli, the latency of the N1m for the sine-wave stimuli was 13 ms [F(2,20) = 23.40, P < 0.001] and 8 ms [F(2,30) = 28.79, P < 0.001] earlier in both the left (109 ms) and right (111 ms) hemisphere.

#### N1m source location

**Figure 4:**
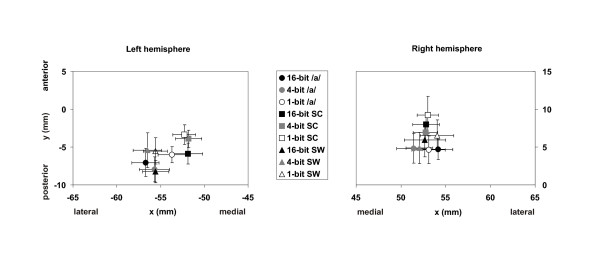
**The N1m ECD location**. Mean N1m ECD location (± SEM) for all subjects and stimuli in the left and right temporal plane (/a/:  vowel /a/; SC: sine-wave composite; SW: sine-wave).

For all stimulus categories, the N1m ECDs were situated in the vicinity of the left and right auditory cortices. As depicted in Fig. 4, the right-hemispheric ECD locations were more anterior than the left-hemispheric ones, which is in line with previous observations [9,10,32]. In the left hemisphere, the sources of the N1m elicited by the sine-wave composite stimuli were approximately 3 mm medial compared to those of the N1m to the vowel and sine-wave stimuli [F(20,2) = 4.75, P < 0.05]. In addition, a significant effect of stimulus degradation was observed on the anterior-posterior axis in the left hemisphere, the sources of the N1m elicited by the 1-bit stimuli being 2 mm anterior to those of the N1m to the 16-bit stimuli [F(2,20) = 4.24, P < 0.05]. In the right hemisphere, the sine-wave composite N1m ECD locations were roughly 3 mm anterior to the sources for vowels [F(2,20) = 5.67, P < 0.05]. In the superior-inferior-dimension, no differences between the ECD locations were observed.

### Gradiometer data

#### P1m amplitude and latency

**Figure 5:**
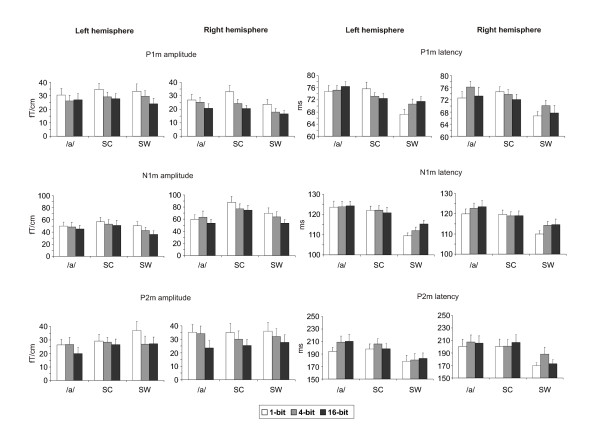
**The effects of stimulus degradation on the P1m, N1m and P2m amplitudes and latencies**. The effects of bit mode on the average P1m, N1m and P2m amplitudes and latencies for each stimulus category (**/a/**:  vowel /a/; SC: sine-wave composite; SW: sine-wave). Error bars indicate SEM.

The peak amplitudes and latencies of the P1m, N1m and P2m responses are shown in Fig. 5. The main effect of stimulus degradation on the amplitude of the P1m was very significant [F(2,26) = 20.97, P < 0.001] and clearly observable in both hemispheres, bit reduction yielding a monotonic increase in the P1m amplitude (22.8, 25.4 & 30.4 fT/cm for the 16-bit, 4-bit & 1-bit modes, respectively). Furthermore, a significant main effect of stimulus type on the P1m amplitude was observed, the sine-wave composite stimuli eliciting a stronger response (28.3 fT/cm) than the other stimulus types (24.2-26.1 fT/cm for tone and speech stimuli, respectively) [F(2,26) = 9.07, P < 0.005]. No hemispheric asymmetries for the P1m amplitude were found [F(1,13) = 3.67, P = n.s.]. However, significant interactions for hemisphere × stimulus type [F(2,26) = 4.59, P < 0.05], stimulus type × bit mode [F(4,52) = 2.93, P < 0.05] and hemisphere × stimulus type × bit mode [F(4,52) = 3.33, P < 0.05] were observed for the P1m amplitude. The average latency of P1m was approximately 5 ms earlier for the sine-wave stimuli (69 ms) than for the other stimulus types (74-75 ms) [F(2,26) = 28.44, P < 0.001]. Moreover, the sine-wave stimuli yielded a bit mode effect on the P1m latency in the left hemisphere (stimulus type × bit mode interaction [F(4,52) = 3.05, P < 0.05]), with the reduction of bit mode resulting in the response peaking earlier (72, 71 & 67 ms for the 16-bit, 4-bit & 1-bit sine-wave).

#### N1m amplitude and latency

The gradiometer data confirmed the results on the N1m response obtained through ECD modeling, including main effects of hemisphere [F(1,13) = 20.30, P < 0.001], stimulus type [F(2,26) = 14.85, P < 0.001] and bit mode [F(2,26) = 12.09, P < 0.001] on the N1m amplitude. However, diverging from the ECD data, additional interactions of hemisphere and stimulus type [F(2,26) = 7.87, P < 0.01] and stimulus type and bit mode [F(4,52) = 2.64, P < 0.05] on the N1m amplitude were found. Post-hoc analysis indicated that the N1m amplitude for the vowel stimuli was less sensitive to the distortion than that for the other stimulus types. These discrepancies between the N1m ECD and gradiometer data are most likely due to the fact that less data was available for the ECD data analyses as a result of inadequate signal-to-noise ratio in five subjects. For the N1m latency, the sine-wave stimuli yielded responses 9 ms earlier (113 ms) than the other stimuli (120-123 ms) [F(2,26) = 44.46, P < 0.001]. In addition, the reduction of stimulus quality resulted in a significant decrease in N1m latency [F(2,26) = 5.17, P < 0.05]. Post-hoc analyses revealed that this effect was evident only for the sine-wave stimuli (stimulus type × bit mode interaction [F(4,52) = 4.01, P < 0.01]), with the peak latency of the N1m ranging from 115 ms (16-bits) to 110 ms (1-bit).

#### P2m amplitude and latency

The P2m response was sensitive to bit mode reduction, its amplitude also increasing with the degree of stimulus degradation (25.1, 30.0 & 33.1 fT/cm for the 16-bit, 4-bit & 1-bit modes, respectively) [F(2,26) = 12.18, P < 0.001]. The peak amplitudes of P2m occurred, on the average, 24 ms earlier for the sine-wave tones (179 ms) than those for the sine-wave composite (202 ms) and speech stimuli (205 ms) [F(2,26) = 23.52, P < 0.001]. The reduction of bit mode resulted in a significant decrease of P2m latency from 196 ms (16-bits) to 190 ms (1-bit) [F(2,26) = 5.14, P < 0.05]. Furthermore, an interaction of hemisphere × stimulus type × bit mode [F(4,52) = 3.04, P < 0.05] was observed for the P2m latency.

### Behavioral measurements

#### Identification accuracy

**Figure 6:**
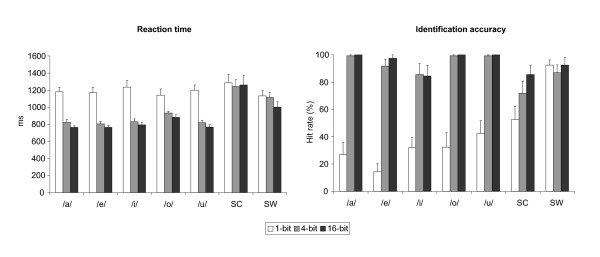
**The behavioral recognition task**. Left: Grand-averaged reaction times for each stimulus and bit mode. Right: Grand-averaged identification accuracy for each stimulus and bit mode. (/a/, /e/, /i/, /o/, /u/: vowel to be recognized; SC: sine-wave composite; SW: sine-wave). Error bars indicate SEM.

The identification accuracy and reaction times in the behavioral recognition experiment are shown in Fig. 6. The vowels and the non-vowel (i.e., the average of the sine-wave composite and the sine-wave) stimuli were identified well beyond the chance level of 11% in the 4-bit (90.6%) and 16-bit (94.3%) conditions. However, in the 1-bit condition, identification accuracy decreased significantly (33.4%) [F(2,30) = 68.80, P < 0.001], except for the sine-wave stimuli (92.5%) [F(2,30) = 1.43, P = n.s.]. Despite this decrement, the accuracy for the 1-bit condition was still above chance level, although the most difficult case, the 1-bit /e/, was identified at a relatively low rate of 14.4%.

#### Reaction times

Reaction times (RTs) for the 1-bit condition, 1183 ms on the average, were significantly increased for all vowel stimuli in relation to the 4- and 16-bit modes (841 ms and 793 ms, respectively) [F(2,30) = 64.57, P < 0.001]. Moreover, RTs were not dependent on vowel identity [F(4,60) = 1.88, P = ns.]. Interestingly, it took 337 ms longer, on the average, to identify the stimuli as a non-vowel than as a specific vowel in the 16-bit and 4-bit conditions (1130 ms and 1177 ms, respectively) [F(6,90) = 16.44, P < 0.001]. There were no statistically significant differences in RTs between the vowel and non-vowel stimuli in the 1-bit condition. Furthermore, for the sine-wave stimuli, the reduction of bit mode resulted in a 130-ms increase in the RTs from 999 ms (16-bit) to 1131 ms (1-bit) [F(2,30) = 5.02, P < 0.02], but no comparable effect was observed for the sine-wave composite stimuli [F(2,30) = 0.07, P = ns.].

## Discussion

The present study investigated the cortical and behavioral processing of speech and non-speech sounds in conditions of decreased signal quality. Unlike in previous research, we distorted the sounds using uniform scalar quantization, which directly reduces the amplitude resolution of the signal waveform thereby introducing signal-dependent, spectrally correlated distortion to the original sound. The results show that degradation of both speech and non-speech sounds amplifies the AEFs bilaterally, indicating that the auditory cortices of both hemispheres are highly sensitive to distortion. The amplitude of the N1m response exhibited hemispheric asymmetry in that the response was significantly stronger and more sensitive to degradation in the right hemisphere than in the left. In addition to the N1m response, we also examined the P1m and P2m responses to investigate the temporal evolution of cortical activation. We found that the degradation amplified these responses as well, the P1m in particular. In contrast to the N1m, the P1m and P2m amplitudes showed no hemispheric asymmetry. The AEF latencies were not systematically affected by the distortion, apart from a small decrease of P1m/N1m/P2m latency for the sine-wave stimuli as the quality of the stimuli was decreased. In addition, the AEF latencies were earlier for the sine-wave than for the speech sound and sine-wave composite stimuli, which corroborates previous findings [32,33]. In the behavioral experiment, stimulus distortion was severe enough to interfere with identification accuracy and reaction times only in the most degraded (1-bit) condition. These findings are congruent with the results of Loizou et al. [4] who demonstrated that for vowel stimuli, an amplitude resolution of four levels (2-bit) is required for successful performance in a recognition task, assuming that spectral resolution is sufficiently high.

The current findings on the processing of speech sounds are in accord with previous results [24], which show that the N1m amplitude is increased in the right hemisphere when acoustical cues of vowels are degraded. The present study indicates, importantly, that this amplifying effect of stimulus distortion is not related to processing of acoustic features of speech in particular, since it was clearly observed with all stimulus types. In fact, the augmentation was somewhat more pronounced with the spectrally simpler, non-speech stimuli than with the speech stimuli. Thus, the increased activation may reflect processing of auditory stimulus features common to both speech and non-speech stimuli. The hemispheric asymmetry observed in [24] was not as distinct in the current data, given that the distortion caused an amplification of the N1m also in the left hemisphere. The hemispheric differences in the N1m amplitude might be related to the changes in spectral structure associated with the stimulus degradation. The larger distortion-related increase of the N1m amplitude in the right hemisphere might reflect an increase in spectral processing caused by the addition of noisy harmonic frequencies to the signal spectrum. This view is corroborated by haemodynamic studies according to which the right-hemispheric auditory cortex is sensitive to spectral variation [26-28], as well as intracortical [34] and AEF [35] findings showing that the right hemisphere is more responsive to the spectral composition of complex tones than the left.

The present results differ from previous observations using additive, uncorrelated noise to degrade speech [16-23] in which the increase of masking noise resulted in decreased and delayed AEP/AEF responses. These differences are interesting, given that both distortion methods render the speech stimuli less intelligible. It seems likely that the increased activation brought about by the USQ manipulation is related to the manner in which this signal distortion technique changes the spectrum of the stimulus. The USQ procedure adds correlated, signal-dependent distortion to the signal over the entire bandwidth, causing an alteration in the distribution and dynamic range of spectral energy. As a result, the balance of spectral energy is shifted towards high frequencies, resulting in a flattening of the spectrum (see Fig. 1H). Further, the quantization process adds new harmonic components to the signal. This is in contrast with the additive (white) noise masking, in which the original spectral harmonics of the intact sound are inundated by an aperiodic masker and due to the linearity of the masking process, no new frequencies are created. Therefore, since both the addition of white noise and the use of the USQ flatten the signal spectrum (and widen the spectral bandwidth in case the undistorted sound has a narrowband spectrum), the most conspicuous spectral difference between the resulting signal spectra is, indeed, the amount of harmonic components. Consequently, one could speculate that the presence of a regular, harmonic spectral structure could be the most relevant feature which induces stronger activation in the brain compared to that elicited by sounds with a random spectral structure. This explanation gains support from recent studies showing that the auditory cortex is sensitive to the periodicity of speech [9,36,37], with periodic stimuli yielding stronger responses than aperiodic ones. Similar results have also been obtained using non-speech sounds, with the presence of a periodic structure leading to enhanced activation [38,39]. The above observations are in accord with the current findings, considering that the quantization adds periodic components to the signal over the whole spectral bandwidth. Taken together, it seems plausible that the auditory cortex is sensitive to harmonic regularities in the sound spectrum, extending even to noisy harmonics.

It could be argued, though, that in the case of the spectrally simpler non-speech stimuli, the increase in spectral bandwidth caused by the distortion could explain the observed AEF augmentation effects regardless of the amount of harmonic components in the signal spectrum. Indeed, the quantization procedure increases the spectral bandwidth of narrowband sounds, for example the sine-wave, by creating new frequency components to the signal. Previous studies have indicated that an increase in spectral bandwidth of tonal, periodic sounds amplifies the N1m response [40]. However, a number of observations speak against an explanation of the N1m enhancement being based on an increase of spectral bandwidth independently of the spectral structure of the stimulus. For instance, in the current study, an increase in the amount of noisy harmonics caused by stimulus distortion amplified the N1m even when the spectral bandwidth remained unchanged (see Fig. 1G &1H, the vowel stimuli). Secondly, in the present observations, the undistorted sine-wave composite stimuli elicited a stronger N1m response than the undistorted vowel, despite having a narrower spectral bandwidth. Thirdly, a number of previous studies suggest that increasing the bandwidth of sounds with a flat, random spectral structure can *decrease *the amplitudes of auditory evoked responses. Soeta et al. [41], for example, demonstrated that the amplitude of the N1m decreased with increasing bandwidth of stochastic noise. Furthermore, Lütkenhöner et al. [39] found that a band-pass random noise elicited a weaker N1m response than a pure tone of narrower bandwidth. In addition, Seither-Preisler et al. [42] did not find any systematic change in the N1m amplitude accompanied with variation in spectral bandwidth of stochastic noise. Similar effects on AEPs have also been documented using continuous masking noise [16,17], the increase in noise bandwidth resulting in diminished AEP responses to speech sounds. Taken together, it seems plausible that the spectral structure of the stimulus has an effect on the related brain responses when the spectral bandwidth of the stimulus is modified. More specifically, it appears that a random frequency spectrum yields decreased responses when the spectral bandwidth is increased while an increase in spectral bandwidth of harmonic components results in amplified AEP/AEFs [24,40]. Thus, we propose that the increase in noisy harmonic components over a wider bandwidth as a result of the quantization procedure could be the main determinant of the observed amplification of the AEF responses.

It might be suggested also that the flattening of the signal spectrum and the relative amplification of higher frequency components would account for the AEF enhancement observed in the present experiment. While it is possible that spectral flattening does have effects on AEF amplitudes, it seems that these effects are to some extent dependent on the degree of harmonic regularity in the signal spectrum. For instance, Palomäki et al. [43] found that wide-band noise bursts elicited substantially smaller N1m responses than vowels and pseudo-vowels even though the noise stimuli had a flat spectrum and thus the high frequencies were significantly emphasized compared to the other stimuli. Hence, it might well turn out that a random increase in high spectral frequencies does not enlarge AEFs whereas an increase in high harmonics yields enhanced AEFs [cf [40]].

The sine-wave stimuli elicited the AEF responses 10 ms earlier than the sine-wave composite and the vowel stimuli in the current experiment. As the sine-wave composite and speech sound contained energy at high frequencies, cochlear travelling wave delays which result in a faster auditory nerve response to high than low frequencies [44] would not seem to account for the cortical latency variations. Rather, these variations may reflect a non-linear relationship between the activity of the cochlea and that of auditory cortex: Roberts and Poeppel [45] demonstrated using pure tones that the N1m latency is shortest to mid-range frequencies and significantly delayed for low and high frequencies. The presence of high frequencies in the speech stimuli may thus have contributed to the observed 10-ms delay in response latency.

The present findings raise interesting questions for future research. For instance, in order to find out why earlier studies have demonstrated decreased and delayed AEP/AEF responses to masked speech stimuli [16-23], the effects of continuous and transient masking of speech should be examined. Transient masking refers to a situation where the distortion is only present simultaneously with the stimulus. It might turn out that the AEP/AEF decrements observed in the above studies arise from the use of continuous masking noise instead of the transient acoustic distortion employed in the present study. Continuous noise causes neuronal adaptation in the auditory cortex, which leads to attenuated and delayed AEP/AEF responses for the masked stimuli: When continuous uncorrelated masking noise is introduced, the intensity of the masked stimuli has to be elevated to produce N1m response amplitudes and latencies equal to those measured in the case of unmasked stimuli [46,47]. The adaptation effects of random noise masking on the N1-P2 response can be clearly observed also when an ongoing noise stimulus becomes periodic and *vice versa *[48]. To obtain an overall picture of the brain dynamics related to the perception of degraded speech, the effects of spectrally correlated distortion and uncorrelated additive noise using both continuous and transient masking on AEF responses should be investigated in the future. These studies might reveal why the introduction of spectrally correlated, signal-dependent distortion to a sound results in a substantial increase in cortical activity while additive, uncorrelated random noise does not, considering that both maskers interfere similarly with the accurate identification of the signal.

## Conclusions

The data presented here suggest that the activation of human auditory cortex as indexed by the amplitude of the AEFs is highly dependent on acoustic degradation of speech and non-speech sounds. More specifically, distorting sounds by reducing the amplitude resolution of the stimulus waveform resulted in amplified AEFs with no systematic changes in response latencies. The N1m amplitudes were asymmetric in that the responses were stronger and more sensitive to distortion in the right hemisphere than the left. We propose that the observed enhancement of cortical activity is related to the distortion caused by the decrease of the amplitude resolution of the signal, in particular the resulting addition of noisy harmonics over the entire bandwidth of the signal spectrum. Taken together, the present results demonstrate that differences in the spectral structure of signal distortion can have substantial effects on brain responses while the perceptual identification of the signal is compromised in a similar manner.

## Abbreviations

AEF: auditory evoked field; AEP: auditory evoked potential; ANOVA: analysis of variance; ECD: equivalent current dipole; EEG: electroencephalography; F0: fundamental frequency; MEG: magnetoencephalography; RT: reaction time; USQ: uniform scalar quantization.

## Authors' contributions

HT, PA, PM and IM designed the experimental setup of the study, and PA prepared the auditory stimuli. IM acquired the data, performed the statistical analysis and prepared the manuscript. All authors participated in the writing process, and have approved the final version of the manuscript.

## References

[B1] Lippmann RP Speech recognition by machines and humans Speech Comm 1997 22 1 15 10.1016/S0167-6393(97)00021-6

[B2] Drullman R Temporal envelope and fine structure cues for speech intelligibility J Acoust Soc Am 1995 97 585 592 10.1121/1.413112 7860835

[B3] Loizou PC Dorman M Tu Z On the number of channels needed to understand speech J Acoust Soc Am 1999 106 2097 2103 10.1121/1.427954 10530032

[B4] Loizou PC Dorman M Poroy O Spahr T Speech recognition by normal-hearing and cochlear implant listeners as a function of intensity resolution J Acoust Soc Am 2000 108 2377 2387 10.1121/1.1317557 11108378

[B5] Shannon R Zeng F-G Kamath V Wygonski J Ekelid M Speech recognition with primarily temporal cues Science 1995 270 303 304 10.1126/science.270.5234.303 7569981

[B6] Saberi K Perrott DR Cognitive restoration of reversed speech Nature 1999 398 760 10.1038/19652 10235257

[B7] Xu L Pfingst BE Spectral and temporal cues for speech recognition: Implications for auditory prostheses Hear Res 2008 242 132 140 10.1016/j.heares.2007.12.010 18249077 PMC2610393

[B8] Studebaker GA Taylor R Sherbecoe RL The effect of noise spectrum on speech recognition performance-intensity functions J Speech Hear Res 1994 37 439 448 8028326 10.1044/jshr.3702.439

[B9] Alku P Sivonen P Palomäki K Tiitinen H The periodic structure of vowel sounds is reflected in electromagnetic brain responses Neurosci Lett 2001 298 25 28 10.1016/S0304-3940(00)01708-0 11154827

[B10] Diesch E Eulitz C Hampson S Ross B The neurotopography of vowels as mirrored by evoked magnetic field measurements Brain and Language 1996 53 143 168 10.1006/brln.1996.0042 8726531

[B11] Mäkelä AM Alku P Tiitinen H The auditory N1m reveals the left-hemispheric representation of vowel identity in humans Neurosci Lett 2003 353 111 114 10.1016/j.neulet.2003.09.021 14664913

[B12] Mäkelä AM Alku P Mäkinen V Tiitinen H Glides in speech fundamental frequency are reflected in the auditory N1m response NeuroReport 2004 15 1205 1208 10.1097/00001756-200405190-00025 15129175

[B13] Mäkelä AM Alku P May PJC Mäkinen V Tiitinen H Left-hemispheric brain activity reflects formant transitions in speech sounds NeuroReport 2005 16 549 553 10.1097/00001756-200504250-00006 15812305

[B14] Obleser J Elbert T Lahiri A Eulitz C Cortical representation of vowels reflects acoustic dissimilarity determined by formant frequencies Cogn Brain Res 2003 15 207 213 10.1016/S0926-6410(02)00193-3 12527095

[B15] Poeppel D Phillips C Yellin A Rowley HA Roberts TPL Marantz A Processing of vowels in supratemporal auditory cortex Neurosci Lett 1997 221 145 148 10.1016/S0304-3940(97)13325-0 9121685

[B16] Martin BA Sigal A Kurtzberg D Stapells DR The effects of decreased audibility produced by high-pass noise masking on cortical event-related potentials to speech sounds /ba/ and /da/J Acoust Soc Am 1997 101 1585 1599 10.1121/1.418146 9069627

[B17] Martin BA Stapells DR Effects of low-pass noise masking on auditory event-related potentials to speech Ear Hear 2005 26 195 213 10.1097/00003446-200504000-00007 15809545

[B18] Whiting KA Martin BA Stapells DR The effect of broadband noise masking on cortical event-related potentials to speech sounds /ba/ and /da/Ear Hear 1998 19 218 231 10.1097/00003446-199806000-00005 9657596

[B19] Muller-Gass A Marcoux A Logan J Campbell KB The intensity of masking noise affects the mismatch negativity to speech sounds in human subjects Neurosci Lett 2001 299 197 200 10.1016/S0304-3940(01)01508-7 11165769

[B20] Kozou H Kujala T Shtyrov Y Toppila E Starck J Alku P Näätänen R The effect of different noise types on the speech and non-speech elicited mismatch negativity Hear Res 2005 199 31 39 10.1016/j.heares.2004.07.010 15574298

[B21] Kaplan-Neeman R Kishon-Rabin L Henkin Y Muchnik C Identification of syllables in noise: Electrophysiological and behavioral correlates J Acoust Soc Am 2006 120 926 933 10.1121/1.2217567 16938980

[B22] Shtyrov Y Kujala T Ahveninen J Tervaniemi M Alku P Ilmoniemi RJ Näätänen R Background acoustic noise and the hemispheric lateralization of speech processing in the human brain: magnetic mismatch negativity study Neurosci Lett 1998 2512 141 144 10.1016/S0304-3940(98)00529-1 9718994

[B23] Shtyrov Y Kujala T Ilmoniemi RJ Näätänen R Noise affects speech-signal processing differently in the cerebral hemispheres NeuroReport 1999 10 2189 2192 10.1097/00001756-199907130-00034 10424696

[B24] Liikkanen LA Tiitinen H Alku P Leino S Yrttiaho S May PJC The right-hemispheric auditory cortex in humans is sensitive to degraded speech sounds NeuroReport 2007 18 601 605 10.1097/WNR.0b013e3280b07bde 17413665

[B25] Cattermole KW Principles of pulse code modulation 1969 London: Iliffe Books

[B26] Zatorre R Belin P Spectral and temporal processing in human auditory cortex Cereb Cortex 2001 11 946 953 10.1093/cercor/11.10.946 11549617

[B27] Jamison HL Watkins KE Bishop DVM Matthews PM Hemispheric specialization for processing auditory nonspeech stimuli Cereb Cortex 2006 16 1266 1275 10.1093/cercor/bhj068 16280465

[B28] Obleser J Eisner F Kotz SA Bilateral speech comprehension reflects differential sensitivity to spectral and temporal features J Neurosci 2008 28 8116 8124 10.1523/JNEUROSCI.1290-08.2008 18685036 PMC6670773

[B29] Alku P Tiitinen H Näätänen R A method for generating natural-sounding speech stimuli for cognitive brain research Clin Neurophysiol 1999 110 1329 1333 10.1016/S1388-2457(99)00088-7 10454267

[B30] Carlson AB Communication systems: An introduction to signals and noise in electrical communication 1986 New York: McGraw-Hill

[B31] Hämäläinen M Hari R Ilmoniemi RJ Knuutila J Lounasmaa OV Magnetoencephalography - theory, instrumentation, and applications to noninvasive studies of the working human brain Rev Mod Phys 1993 65 413 497 10.1103/RevModPhys.65.413

[B32] Tiitinen H Sivonen P Alku P Virtanen J Näätänen R Electromagnetic recordings reveal latency differences in speech and tone processing in humans Cogn Brain Res 1999 8 355 363 10.1016/S0926-6410(99)00028-2 10556611

[B33] Eulitz C Diesch E Pantev C Hampson S Elbert T Magnetic and electric brain activity evoked by the processing of tone and vowel stimuli J Neurosci 1995 15 2748 2755 7722626 10.1523/JNEUROSCI.15-04-02748.1995 PMC6577747

[B34] Liégeois-Chauvel C Giraud K Badier JM Marquis P Chauvel P Intracerebral evoked potentials in pitch perception reveal a functional asymmetry of the human auditory cortex Ann N Y Acad Sci 2001 930 117 132 11458823 10.1111/j.1749-6632.2001.tb05728.x

[B35] Schneider P Sluming V Roberts N Scherg M Goebel R Specht HJ Dosch HG Bleeck S Stippich C Rupp A Structural and functional asymmetry of lateral Heschl's gyrus reflects pitch perception preference Nat Neurosci 2005 8 1241 1247 10.1038/nn1530 16116442

[B36] Tiitinen H Mäkelä AM Mäkinen V May PJC Alku P Disentangling the effects of phonation and articulation: Hemispheric asymmetries in the auditory N1m response of the human brain BMC Neurosci 2005 6 62 70 10.1186/1471-2202-6-62 16225699 PMC1280927

[B37] Yrttiaho S Tiitinen H May PJC Leino S Alku P Cortical sensitivity to periodicity of speech sounds J Acoust Soc Am 2008 123 2191 2199 10.1121/1.2888489 18397025

[B38] Soeta Y Nakagawa S Tonoike M Auditory evoked magnetic fields in relation to iterated rippled noise Hear Res 2005 205 256 261 10.1016/j.heares.2005.03.026 15953534

[B39] Lütkenhöner B Seither-Preisler A Seither S Piano tones evoke stronger magnetic fields than pure tones or noise, both in musicians and non-musicians NeuroImage 2006 30 927 937 16337814 10.1016/j.neuroimage.2005.10.034

[B40] Seither-Preisler A Krumbholz K Lütkenhöner B Sensitivity of the neuromagnetic N100 m deflection to spectral bandwidth: a function of the auditory periphery? Audiol Neuro-otol 2003 8 322 37 10.1159/000073517 14566103

[B41] Soeta Y Nakagawa S Tonoike M Auditory evoked fields in relation to bandwidth variations of bandpass noise Hear Res 2005 202 47 54 10.1016/j.heares.2004.09.012 15811698

[B42] Seither-Preisler A Patterson RD Krumbholz K Seither S Lütkenhöner B From noise to pitch: Transient and sustained responses of the auditory evoked field Hear Res 2006 218 50 63 10.1016/j.heares.2006.04.005 16814971

[B43] Palomäki KJ Tiitinen H Mäkinen V May PJC Alku P Cortical processing of speech sounds and their analogues in a spatial auditory environment Cogn Brain Res 2002 14 294 299 10.1016/S0926-6410(02)00132-5 12067702

[B44] Patterson RD The sound of a sinusoid: spectral models J Acoust Soc Am 1994 96 1409 1418 10.1121/1.410285

[B45] Roberts TPL Poeppel D Latency of auditory evoked M100 as a function of tone frequency NeuroReport 1996 7 1138 1140 10.1097/00001756-199604260-00007 8817518

[B46] Morita T Nobuya F Nagamine T Hiraumi H Naito Y Shibasaki H Ito J Effects of continuous masking noise on tone-evoked magnetic fields in humans Brain Res 2006 1087 151 158 10.1016/j.brainres.2006.03.004 16626668

[B47] Billings CJ Tremblay KL Stecker GC Tolin WM Human evoked cortical activity to signal-to-noise ratio and absolute signal level Hear Res 2009 254 15 24 10.1016/j.heares.2009.04.002 19364526 PMC2732364

[B48] Martin BA Boothroyd A Cortical, auditory, event-related potentials in response to periodic and aperiodic stimuli with the same spectral envelope Ear Hear 1999 20 33 44 10.1097/00003446-199902000-00004 10037064

